# Cardiovascular Complications of Hematopoietic Stem Cell Transplantation

**DOI:** 10.1007/s11936-016-0447-9

**Published:** 2016-02-24

**Authors:** Anne Blaes, Suma Konety, Peter Hurley

**Affiliations:** Division of Hematology/Oncology/Transplantion, University of Minnesota, 420 Delaware Street, S.E., MMC 480, Minneapolis, MN 55455, USA; Division of Cardiology, University of Minnesota, Minneapolis, MN USA

**Keywords:** Stem cell transplantation, Cardiac complications, Congestive heart failure, Arrhythmias, Survivors

## Abstract

Survivors of hematopoietic stem cell transplant (HSCT) are at significant risk for cardiac disease and cardiac complications. While there may be cardiac complications during the acute period of HSCT, long-term survivors remain at risk for cardiovascular disease at a rate at least fourfold higher than the general population. Aggressive screening for cardiac risk factors such as diabetes, hypertension, and arrhythmias is warranted pretransplant. For those with risk factors, particularly a history of cardiovascular disease or atrial fibrillation, cardiology consultation is warranted in the pretransplantation period. Aggressive screening for cardiac risk factors such as diabetes, hypertension, and hyperlipidemia is warranted in HSCT survivors as well; early and aggressive treatment of left ventricular dysfunction is warranted. Collaboration between hematology/oncology and cardiology through a cardio-oncology clinic is an optimal way to help manage these patients.

## Introduction

Hematopoietic stem cell transplantation (HSCT) is a potential cure for various hematologic malignancies, and rarely non-malignant conditions. Since 1980 when HSCT first began as a lifesaving therapy, the number of allogeneic transplantations has continuously risen with over 320,000 individuals having received a stem cell transplantation by 2013.[1] HSCT consists of the administration of myelosuppressive chemotherapy with or without total body irradiation followed by infusion of a hematopoietic stem cell graft that induces an immunologic response on malignant cells. The preparative regimen and the immune response are the major sources of treatment-related toxicity. Identifying patients fit enough to tolerate such toxicity and anticipating and managing expected complications are key aspects to improving HSCT outcomes.

## Acute cardiac complications

Cardiovascular disease and cardiovascular complications are one of the most common complications associated with HSCT. These complications can occur both acutely within the first 100 days as well as long-term, many years after the initial transplantation period. Prior studies report that 9–27 % of persons receiving HSCT develop arrhythmias,[2•] while other more recently published single institutions have reported incidences as low as 1 %.[3] The most common arrhythmias include atrial fibrillation, atrial flutter, and supraventricular tachycardia. Risk factors for the development of arrhythmias include the following: older age, prior anthracycline use, a lower ejection fraction at baseline, history of arrhythmias, baseline renal dysfunction, and the presence of premature supraventricular complexes on baseline screening electrocardiogram.[4–6] The effect of these arrhythmias on outcomes was largely unknown until a recent publication evaluated the impact of arrhythmias on HSCT. In examining 104 out of 1177 individuals who underwent HSCT between 1999 and 2009, subjects with an arrhythmia post transplant were more likely to have a longer median hospital stay (32 vs 23 days, *p* < 0.001), a greater probability of an intensive care unit admission (52 vs 7 %, *p* < 0.001), and greater probability of death within 1 year of transplant (41 vs 15 %, *p* < 0.001). In a multivariate analysis, posttransplant arrhythmia was associated with a greater risk for death within a year of transplant [OR 3.5 (2.1–5.9)]. These results have been confirmed in other studies in autologous HCT recipients. Of those treated between 2000 and 2006 who developed arrhythmias during the first 100 days post transplantation, the risk of mortality was 40 % [5].

Other cardiac complications of HSCT include congestive heart failure (CHF), cardiac tamponade, and ventricular arrhythmias resulting in death.[3] While cardiac tamponade and ventricular arrhythmias are rare occurring in <1 % of recipients, CHF remains a concern particularly as the age of individuals undergoing HSCT increases. Most preparative regimens for allogeneic HSCT use cyclophosphamide (CY). High-dose CY can induce myocardial necrosis which clinically presents with dyspnea, tachycardia, hypotension, decreased QRS voltage on electrocardiogram, and pericardial effusion within ten days of drug administration.[7–10] The histology in these cases shows a unique pattern of fibrin microthrombi in capillaries, fibrin strands in the interstitium, and fibrin strands in myocytes.[11] Felt to be dose related with higher rates of heart failure in those receiving doses ≥170 mg/m^2^,[7, 12] heart failure occurred in as many as 28 % of cases of HSCT using older regimens with higher dose CY. With CY dose reductions in contemporary regimens, heart failure appears less frequent.[3, 7, 8, 11] Recent studies suggest congestive heart failure occurs <2 % of the time.[13•]

With an understanding of the greater therapeutic effect on malignancy of the immune response induced by the graft compared to the chemotherapy conditioning, reduced intensity conditioning (RIC) regimens that spare some of the toxicity of a myeloablative preparative regimen have been developed in recent years.[14] Melphalan and fludarabine are agents used in some RIC regimens that have been reported to cause cardiovascular complications. Melphalan, as part of RIC or autologous HSCT regimens, has been associated with atrial fibrillation, atrial flutter, and supraventricular tachycardia in up to 9 % of cases.[5, 15–17] The combination of melphalan and fludarabine has been reported in some case series to cause heart failure ranging in severity from minor and reversible systolic dysfunction to fatal heart failure.[18–20] Cardiovascular complications thus should remain a consideration even for patients undergoing RIC.

The development of RIC made HSCT a treatment option for patients with comorbidities and who are older.[1, 21, 22] How to account for comorbidities in the pretransplant evaluation and how to manage them during the transplantation process remains a challenge for clinicians. Through the HSCT-specific comorbidity index, a cardiac comorbidity (defined as the presence of coronary artery disease, congestive heart failure, myocardial infarction, or an ejection fraction <50 %) is considered as a low-risk comorbidity. Further, recent data suggests that those with an ejection fraction <50 % can still be eligible for HSCT and patients with borderline left ventricular systolic dysfunction can safely undergo HSCT without alterations in overall survival or treatment-related complications.[23] As the population ages and more and more transplants are being done on individuals >65 years old which many have underlying cardiovascular disease at baseline, a complete geriatric assessment (CGA) in these patients may be beneficial.[24, 25] The CGA focuses not only on comorbidities but also includes assessments of dementia, delirium, failure to thrive, incontinence, osteoporosis, falls, polypharmacy, and sarcopenia.[24–26] There are little data available on the role of CGAs in the HSCT population; however, this is quickly changing [27–29].

### Long-term cardiac complications from HSCT

For those individuals who survive the first 100 days of HSCT, HSCT survivors remain at an increased risk for cardiovascular complications many years after transplantation.[13•, 30–33] Single institution studies suggest the 10-year cumulative incidence of hypertension is 37.7 %, diabetes 18.1 %, hyperlipidemia 46.7 %, and having more than one of these cardiac risk factors at 31.4 %.[32] Most of these risks are seen in allogeneic HSCT survivors with the risk being highest in those at an older age, with obesity, and with a history of grade 2–4 acute graft-versus-host disease. The risk of cardiovascular anthracycline-induced cardiomyopathy complications in autologous HSCT survivors, however, also remains of concern in which the cumulative incidence of CHF at 5 years after HSCT of 4.8 %, increasing to 9.1 % at 15 years.[34] Overall, this cardiovascular risk is 4.5-fold higher than that seen in the general population. Prior anthracycline use and chest radiation also increases this risk.

### Treatment of cardiovascular complications of HSCT

#### First 100 days of HSCT

There are little specific data on how to manage systolic dysfunction in patients undergoing HSCT; thus, its management during transplantation should be the same as the general population with beta blockers and ACE inhibitors per the American Heart Association and American College of Cardiology guidelines.[35] Evidence that beta blockers (particularly carvedilol) and ACE inhibitors are effective for preventing anthracycline-induced cardiomyopathy provides further rationale for this practice in oncology patients.[36–39] These medications have also been shown to improve LVEF in subjects with anthracycline-induced cardiomyopathy.[40]

Evaluation of cardiac systolic function by multiple gated acquisition scan (MUGA), echocardiogram, or cardiac magnetic resonance imaging (MRI) is a recommended part of a pre-transplantation evaluation.[41, 42] Attention to any signs of left ventricular dysfunction or premature supraventricular complexes on screening electrocardiogram should be reviewed carefully; consideration of preventative therapy with beta blockers and angiotensin-converting enzyme (ACE) inhibitors should be considered in this population given their higher risk for arrhythmias during transplantation.[37, 38, 40, 43] Referral to a cardio-oncology clinic, for those with any cardiovascular risk factors or prior cardiac disease or arrhythmias, is recommended.[44•, 45, 46]

#### Treatment of long-term cardiac complications from HSCT

Given the increased burden for serious cardiovascular complications following HSCT, a variety of long-term recommendations through the Children’s Oncology Group and the Center for International Blood and Marrow Transplantation have been developed for the treatment of cardiovascular risk factors in HSCT survivors.[47–49] Given these guidelines differ from the cardiovascular screening recommendations of the general population as outlined by the United States Services Preventative Task Force, we have provided an outline of these recommendations below:

- Lifestyle modifications
  - As high as 30 % of cancer survivors, particularly in the younger aged population, smoke.[50] Patients should be counseled to abstain from tobacco use. While tobacco use is associated with secondary cancers, it is also a recognized inducer of cardiac disease.
  - Additional lifestyle modifications including a low fat diet, maintaining a healthy weight, and regular exercise should be part of their counseling.
  - Additionally, the American College of Sports Medicine recommends at least 150 min of moderate exercise per week, regardless of age or treatment used during HSCT.[51]
- Diabetes/impaired glucose tolerance testing
  - With the 10-year cumulative incidence of diabetes being >18 % in HSCT survivors,[32] it is recommended that a fasting glucose be obtained every 3 years after the age of 45 years or earlier if blood pressure >135/80 mmHg.
  - In childhood cancer survivors where the rates of metabolic syndrome are even higher, the Children’s Oncology Group recommends a fasting glucose every 2 years after HSCT, particularly in those who received cranial radiation or total body irradiation as part of their preparative regimen.[48, 52]
- Dyslipidemia (fasting lipids) testing
  - With dyslipidemia occurring in >46 % of HSCT survivors [32] and many HSCT recipients having multiple cardiac risk factors, [5, 13•] fasting lipids should be checked every 5 years starting at age 35 years for men, age 45 years for women, and earlier for those with other cardiovascular risk factors. For higher risk individuals who use tobacco, have diabetes, hypertension, obesity, or a family history of cardiovascular disease, screening should begin at age 20 years.
  - In childhood HSCT survivors, lipids should be checked every 2 years beginning 2 years after HSCT, particularly in those who received cranial radiation or total body irradiation.[13•]
- Electrocardiogram screening
  - While not recommended for the general population (UPSTF), screening with electrocardiogram should be considered for individuals with risk factors for cardiovascular disease, namely amyloidosis, prior chest/mediastinal radiation therapy, preexisting cardiovascular disease. With more than 50 % of patients with amyloidosis also having cardiac involvement, this patient population is particularly at risk for arrhythmias.[47, 49, 53] Monitoring electrocardiograms may be helpful. Additionally, recipients of chest/mediastinal radiation have rates of cardiac disease of 17 % by age 35 years.[54] With cardiac deaths being a large cause of mortality in recipients of chest/mediastinal radiation, screening electrocardiograms may be helpful [47, 49, 54, 55].
  - For childhood HSCT survivors, baseline electrocardiogram is recommended 2 years after the completion of HSCT [13•].
- Cardiac Imaging in Survivors
  - In asymptomatic adults without any cardiac risk factors, cardiac imaging tests such as transthoracic echocardiography, carotid intima-media thickness by ultrasound, stress echocardiography, and cardiac magnetic resonance imaging are not recommended. In individuals, however, with risk factors such as hypertension or diabetes, prior chest radiation, history of amyloidosis, preexisting cardiac disease, baseline electrocardiogram, and transthoracic echocardiography could be considered [49]. Stress echocardiography may also be useful in those who have received prior cardiac mediastinal radiation [56].
  - In children HSCT survivors, the Children’s Oncology Group has specific recommendations for interval of echocardiography based on age of exposure, cumulative anthracycline dose exposure, and/or chest radiotherapy [13•].
  - The frequency of echocardiography screening based on age of cancer diagnosis and treatment, receipt of radiation, and receipt of anthracycline use is not known [41]; aggressive screening to monitor for left ventricular dysfunction with subsequent early pharmacologic intervention has been shown to be cost effective, although research regarding the exact interval of screening is ongoing [57].
  - Ongoing research as to the use of echocardiography with strain and echocardiography with speckle tracking is ongoing [42, 58–60]. Similarly, the use of cardiac MRI as a screening tool for cardiac fibrosis and left ventricular remodeling is currently being studied. While not useful in all HSCT survivors, it may be useful in select high-risk patients [41, 61].
  - MUGA scan is discouraged given the use of radiation.
- Other
  - Screening for arterial disease with ankle brachial index and carotid artery imaging is not recommended for the general HSCT population. Consideration for carotid artery ultrasound imaging 10 years post HSCT should be considered for childhood HSCT survivors as well as those treated with prior mantle radiation [13•, 49].
- Pharmacologic Interventions
  - There is little specific data on how to manage left ventricular dysfunction in patients undergoing HSCT; thus, its management post transplantation should be the same as the general population with beta blockers and ACE inhibitors per the American Heart Association and American College of Cardiology guidelines [35]. Evidence that beta blockers and ACE inhibitors are effective for treating anthracycline-induced cardiomyopathy provides further rational for this practice in these oncology patients [36–38].
  - Similarly, aggressive management of hyperlipidemia and diabetes as per the American Diabetes Association is recommended.
  - Ongoing work suggests treating early left ventricular function with ACE inhibitors or beta blockers may actually be cost-effective [57].
- Cancer Survivorship Clinics
  - Given the elevated risks of cardiac disease in HSCT as well as specific recommendations for surveillance and follow-up care, consultation with a cancer survivorship clinic and receipt of a survivorship care plan (SCP) may be useful in promoting knowledge of cardiac risk as well screening recommendations [55, 62].
  - Finally, for those individuals at highest risk for cardiac disease, such as those with amyloidosis or who also have received prior chest/mediastinal radiation who have also undergone HSCT, referral to a cardio oncology clinic is extremely important. Collaboration between hematology/oncology and cardiology is of critical importance in providing the best evidence based care for recipients for HSCT [43, 44•].

## Conclusion

Survivors of HSCT are at significant risk for cardiac disease and cardiac complications. While there may be cardiac complications during the acute period of HSCT, long-term survivors remain at risk for cardiovascular disease at a rate at least fourfold higher than the general population. Aggressive screening for cardiac risk factors such as diabetes, hypertension, and hyperlipidemia is warranted as is early and aggressive treatment of left ventricular dysfunction. Collaboration between hematology/oncology and cardiology through a cardio-oncology clinic is an optimal way to help manage these patients.
